# Comparative visual ecology of cephalopods from different habitats

**DOI:** 10.1098/rspb.2016.1346

**Published:** 2016-09-14

**Authors:** Wen-Sung Chung, N. Justin Marshall

**Affiliations:** Queensland Brain Institute, The University of Queensland, St Lucia 4072, Australia

**Keywords:** cephalopod, microspectrophotometry, spectral tuning, opsin

## Abstract

Previous investigations of vision and visual pigment evolution in aquatic predators have focused on fish and crustaceans, generally ignoring the cephalopods. Since the first cephalopod opsin was sequenced in late 1980s, we now have data on over 50 cephalopod opsins, prompting this functional and phylogenetic examination. Much of this data does not specifically examine the visual pigment spectral absorbance position (*λ*_max_) relative to environment or lifestyle, and cephalopod opsin functional adaptation and visual ecology remain largely unknown. Here we introduce a new protocol for photoreceptor microspectrophotometry (MSP) that overcomes the difficulty of bleaching the bistable visual pigment and that reveals eight coastal coleoid cephalopods to be monochromatic with *λ*_max_ varying from 484 to 505 nm. A combination of current MSP results, the *λ*_max_ values previously characterized using cephalopod retinal extracts (467–500 nm) and the corresponding opsin phylogenetic tree were used for systematic comparisons with an end goal of examining the adaptations of coleoid visual pigments to different light environments. Spectral tuning shifts are described in response to different modes of life and light conditions. A new spectral tuning model suggests that nine amino acid substitution sites may determine the direction and the magnitude of spectral shifts.

## 1. Introduction

No other creature in the animal kingdom can beat the versatility of coleoid cephalopod dynamic coloration and camouflage [1]. Although coleoids can display complex colour patterns on their skin, behavioural, physiological and anatomical studies indicate that most are colour-blind [2–4]. For instance, Messenger [2] demonstrated that the common reef octopus, *Octopus vulgaris*, was capable of recognizing objects based on brightness levels, but failed in all colour discrimination trials. Similar results were found in the common European cuttlefish, *Sepia officinalis*, where animals showed different body patterns to match the background using intensity instead of wavelength cues [3,4].

Morphological examinations demonstrate that most coleoids apparently possess a single photoreceptor type and that these are in some ways similar to the rod-dominant retina of deep-sea fish [5,6]. Coleoid visual pigments are, however, embedded in microvillar type photoreceptors, as also found in other invertebrates such as crustaceans or insects [7–9]. The visual pigment rhodopsin consists of a chromophore linked to an opsin, and their specific combinations determine the peak of the spectral absorbance (*λ*_max_) and the shape of the absorbance spectrum of the visual pigment [10–14]. Two methods have been commonly used to determine the *λ*_max_ of cephalopod visual pigment: spectrophotometry of detergent extracts of visual pigment (ESP) and photoreceptor microspectrophotometry (MSP) [8,13,15,16]. It is clear that most cephalopods have only one blue-green-sensitive visual pigment where the range of *λ*_max_ value is similar to the rod photoreceptors in fish (approx. *λ*_max_ 470–505 nm; table 1) [10,11,13,16,21–23]. While most cephalopods use retinal (A1 chromophore) to make visual pigment, a small group of mid-water squid (enoploteuthid and bathyteuthid) construct multiple visual pigments using different chromophores, 3-dehydroretinal (A2 chromophore) and 4-hydroxyretinal (A4 chromophore), to expand their spectral range and potentially distinguish colours [8,15].

**Table 1.** Lists of spectral sensitivity of cephalopod and available opsin sequences from GenBank. The capital E indicates *λ*_max_ obtained using ESP; M indicates that using MSP; C indicates coastal waters; P, pelagic waters. The number after *λ*_max_ value and living depth indicates the reference.

animals	opsin GenBank accessing no.	*λ*_max_ (nm)	habitat (living depth range)	animals	opsin GenBank accessing no.	*λ*_max_ (nm)	habitat (living depth range)
Nautilida				Spirulida			
* Nautilius pompilius*^a^	LC021433	E467 [17]	P, 60–750 m [18]	* Spirula spirula*	AY545183	—	P, 300–1750 m [18]
Vampyroteuthida				Bathyteuthida			
* Vampyroteuthis infernalis*	AY545163	—	P, 600–1200 m [19]	* Bathyteuthis berryi*	AY616912	—	P, 800–1200 m [20]
Octopoda				* Bathyteuthis magister*	—	E484 [21]	P, —
* Callistoctopus dierythraeus*	—	M487^b^	C, 0–78 m [19]	Myopsida			
* Callistoctopus ornatus*	AY616926	—	C, 0–10 m [19]	* Alloteuthis subulata*^a^	ZA9108	E499 [11]	C, 50–500 m [20]
* Enteroctopus dofleini*^a^	X07797	E480 [21]	C, 0–1500 m [19]	* Heterololigo bleekeri*	KF854109	E494 [21]	C, 0–150 m [20]
* Eledone moschata*	—	E470 [16]	C, 10–300 m [19]	* Loligo forbesii*^a^	X56788	E494 [11]	C, 50–700 m [20]
* Hapalochlaena maculosa*	AY545171	M485^b^	C, 0–50 m [19]	* Loligo pealeii*^a^	AY450853	E493 [22]	C, 0–390 m [20]
* Octopus australis*	—	M485^b^	C, 0–134 m [19]	* Loliolus japonica*	—	E496 [21]	C, —
* Octopus minor*	—	E480 [21]	C, —	* Loliolus beka*	—	E496 [21]	C, —
* Octopus bimaculoides*^a^	XM_014927502	—	C, 0–50 m [19]	* Sepioteuthis australis*	AY616917	—	C, 0–70 m [20]
* Octopus ocellatus*	—	E480 [21]	C, 0–100 m [19]	* Sepioteuthis lessoniana*	AY616918	M503^b^	C, 0–100 m [20]
* Octopus tetricus*	—	M487^b^	C, 0–60 m [19]	* Uroteuthis edulis*	—	E491 [21]	C, 30–170 m [20]
* Octopus vulgaris*^a^	KR90290	E475 [22]	C, 0–100 m [19]	Sepiolida			
Idiosepiida				* Euprymna morsei*	—	E494 [21]	C, —
* Idiosepius notoides*	AY545181	M493^b^	C, 0–20 m [18]	* Euprymna scolopes*^a^	EU344773	—	C, 0–200 m [18]
* Idiosepius paradoxus*^a^	LC021434	—	C, 0–20 m [18]	* Euprymna tasmanica*	AY617049	M499^b^	C, 0–80 m [18]
Oegopsida				* Sepiola atlantica*	—	E492 [16]	C, 3–150 m [18]
* Enoploteuthis chunii*	—	E484 [21]	P, 50–300 m [20]	* Sepiola* sp.	AY545182	—	C, —
* Histioteuthis meleagroteuthis*	—	E480 [16]	P, 210–1250 m [20]	Sepiida			
* Histioteuthis oceanica*	AY617053	—	P, —	* Metasepia tullbergi*	AY616925	—	C, 3–86 m [18]
* Illex coindetii*	AY617062	—	P, 50–600 m [20]	* Sepiella japonica*	—	E504 [21]	C, 0–50 m [18]
* Mastigoteuthid hjorti*	—	E482 [16]	P, —	* Sepia esculenta*	—	E490 [21]	C, 10–100 m [18]
* Ommastrephes bartramii*	AY616915	E482 [21]	P, 0–500 m [20]	* Sepia latimanus*^a^	KR107049	—	C, 0–30 m [18]
* Pterygioteuthis microlampas*	AY616913	—	P, —	* Sepia lycidas*	—	E491 [21]	C, 15–100 m [18]
* Pyroteuthis margaritifera*	—	E480 [16]	P, 50–800 m [20]	* Sepia officinalis*^a^	AF000947	E493 [22]	C, 50–200 m [18]
* Todarodes pacificus*^a^	X70498	E482 [21]	P, 50–500 m [20]	* Sepia plangon*	—	M499^b^	C, 0–83 m [18]
* Watasenia scintillans*	—	E470 [21] E484 [21] E500 [21]	P, 100–600 m [20]	* Sepia pharaonis*	AY616924	—	C, 10–130 m [18]

^a^Species name indicates that the full-length opsin transcript is available.

^b^The current MSP results.

Visual pigments are a frequently used model system for learning how protein variations alter sensory function and phenotype and mediate the requirements of vision [6,9–11,24]. Adaptive variation in visual pigment and spectral sensitivities are of particular interest in respect to visual ecology and molecular evolution in both vertebrates and invertebrates [9–11,24,25]. A growing number of cephalopod opsin sequences, particularly coleoids, are available in the GenBank database [11,26–28]; however, lack of *λ*_max_ information in most sequenced coleoid opsins makes available molecular data of limited use. Therefore, the phylogenetic tree of coleoid opsin cannot be reliably used to reflect functional adaptations with respect to light environments and modes of life.

Coleoid cephalopods are attractive for studying the evolution of vision as they have camera-like eyes, sharing many similarities in optics, anatomy and function with fish, while having evolved these parallels through convergence [5,26]. In contrast with fish rhodopsins, which have been extensively investigated [10,29], knowledge of the comparative functional adaption of coleoid visual pigments remains sparse. Our goal in this study was to investigate spectral adaptation in different habitats. We tested the hypothesis that habitat and corresponding light conditions drive the spectral tuning of coleoid cephalopods.

In order to achieve this, first, a new MSP protocol was developed to make direct measurement of spectral sensitivity in eight species of coastal coleoids and characterize the spectral sensitivity of photoreceptors across many retinal regions. Second, the opsin phylogenetic trees and the relationship between *λ*_max_ and environmental characters revealed that spectral tuning occurs in the decapodiform coleoids, whereas octopods do not show similar adaptation. The spectral sensitivity of these decapodiforms shows depth-dependent spectral changes linked to their dwelling realms and modes of life. Furthermore, with opsin sequence alignments and multiple comparisons of amino acid replacements, we proposed that nine amino acid substitution sites are likely to determine the direction and the magnitude of spectral shifts in coleoid visual pigments.

## 2. Material and methods

### (a) Animals

Five coastal coleoids—*Idiosepius notoides*, *Euprymna tasmanica, Sepioteuthis lessoniana*, *Sepia plangon* and *Octopus australis*—were collected using a seine net (water depth 1–3 m) close to Moreton Bay Research Station, Stradbroke Island, Queensland. Another three octopus species—*Callistoctopus dierythraeus*, *Hapalochlaena maculosa* and *Octopus tetricus*—were collected in Moreton Bay (water depth 5–10 m) by a local shellfish supplier (Queensland Sustainable Sealife). Habitats and living depth range of selected animals are listed in table 1. Animals were maintained in a 400 l tank of artificial seawater lit by standard daylight fluorescent tubes on a 12 L : 12 D cycle and used for MSP within a week of capture.

### (b) Retinal preparation

Hubbard & St George [13] found that the photochemical reactions of squid rhodopsin and photo-products were pH-dependent *in vitro*. Using this feature, different mounting solutions were employed to examine the photosensitivities of coleoid photoreceptors as follows: (i) standard mounting solution (0.1 M phosphate buffer saline (PBS) (17–515DPBS, Lonza, USA) mixed with 6% sucrose, pH 7.4); (ii) alkaline mounting solution (pH of standard mounting solution is adjusted to 10 using 0.1 M NaOH); (iii) diluted hydroxylamine solution (50% w/v hydroxylamine solution (Merck, Germany) diluted using 0.1 M PBS to 25%, 10% and 1%).

Animals were dark-adapted overnight prior to the retinal preparation for MSP. The specimen was anaesthetized in cold seawater mixed with 2% MgCl_2_ and then decapitated. Under dim red illumination, eyecups were removed and equally divided into four quadrants. Three retinal samples of each quadrant were selected and embedded in 10% sucrose mixed with the optimal cutting temperature compound (OCT) (Tissue-Tek, Sakura Finetek, USA) for cryosectioning at −20°C also under dim red illumination. Transverse sections of the retina (12 µm thickness) were collected with a coverslip (22 × 64 mm #1, Menzel-Glaser, Germany). With a drop of mounting solution, the sample was covered with a circular glass coverslip (10 mm diameter #0, Chance Propper, UK) and sealed with silicone vacuum grease.

### (c) Microspectrophotometry operation and data analysis

Operation of MSP followed a standard protocol developed for vertebrate or invertebrate photoreceptors [30,31]. The measuring light beam was set to a size of around 2 × 15 µm and placed parallel to the long axis of the rhabdome. Baseline and sample scans were made from tissue-free and cellular regions of the preparation, respectively. Subsequently, the visual pigments were bleached using a white light beam. The bleaching process was repeated in some cases until effective visual pigment bleaching occurred. Best-fit visual pigment nomograms were used to determine the *λ*_max_ of each sample following the methods developed by MacNichol [32], Govardovskii *et al.* [12] and Hart *et al.* [31]. Data from three or more individual measurements were averaged (electronic supplementary material, table S1).

A hypsochromic shift (blue shift, approx. 5 nm) introduced by the visual pigment purification process and detergent extraction has previously been reported in measurement of *λ*_max_ in several animals including cephalopods [7,12]. In an effort to determine the variance in coleoid *λ*_max_ between two methods, comparisons of ESP and the current MSP data across four coastal coleoid groups were then used to determine the offset value for further analyses.

### (d) Phylogenetic analyses

Determination of cephalopod phylogenetic relationships followed the classification published by Allcock *et al.* [33]. In order to cover the major lineages of cephalopod, 28 sequenced opsins from 10 orders were downloaded from GenBank (table 1) for analyses, including representatives inhabiting different light environments [18–20]. All these selected samples include 12 full-length opsin transcripts and 16 partial opsin transcripts (more than 198 amino acids in the transmembrane region). Alignments of opsin were constructed from amino acid sequences using the multiple sequence alignment (MUSCLE) method with MEGA 6 (molecular evolutionary genetics analysis program v. 6.06-Mac) [34] and then refined visually using numerous highly conserved amino acid sites. *Nautilus pompilius* was used as the outgroup. Two types of opsin trees were constructed: (i) using 12 full-length opsin transcripts and (ii) using partial opsin transcripts by 28 species. The phylogenetic tree of cephalopod opsin was generated by the maximum-likelihood method and the bootstrap confidence values (1000 replicates) were calculated with MEGA 6 [34].

The phylogenetic signal was estimated with Pagel's *λ* using the package CAPER v. 0.5.2 of the software program R v. 3.2.3 as implemented in the RStudio v. 0.99.891 (2016). The relationship between *λ*_max_ and environmental characters (electronic supplementary material, table S3) was determined using the phylogenetic generalized least-squares (PGLS) method with the CAPER package in RStudio.

### (e) Sequence analyses and site predictions responsible for spectral tuning

The numbers of amino acid sites which differed among 12 full-length cephalopod opsins were summed in four ways as follows: (i) the total number of sites that differed; (ii) the number of differences occurring within the transmembrane regions; (iii) the number of difference at sites within the chromophore binding pocket; (iv) the number of sites in the chromophore binding pocket which differed in amino acid polarity. Potential functional amino acid substitutions were searched for by comparing known key tuning sites [11]. In addition, substitutions differing in amino acid polarity in the chromophore binding pocket were identified by multiple comparisons of amino acid alignments and then inspected using the function of estimate position-by-position rates in MEGA 6 [34].

## 3. Results

### (a) Photochemistry reactions under different mountants

**Figure 1.**
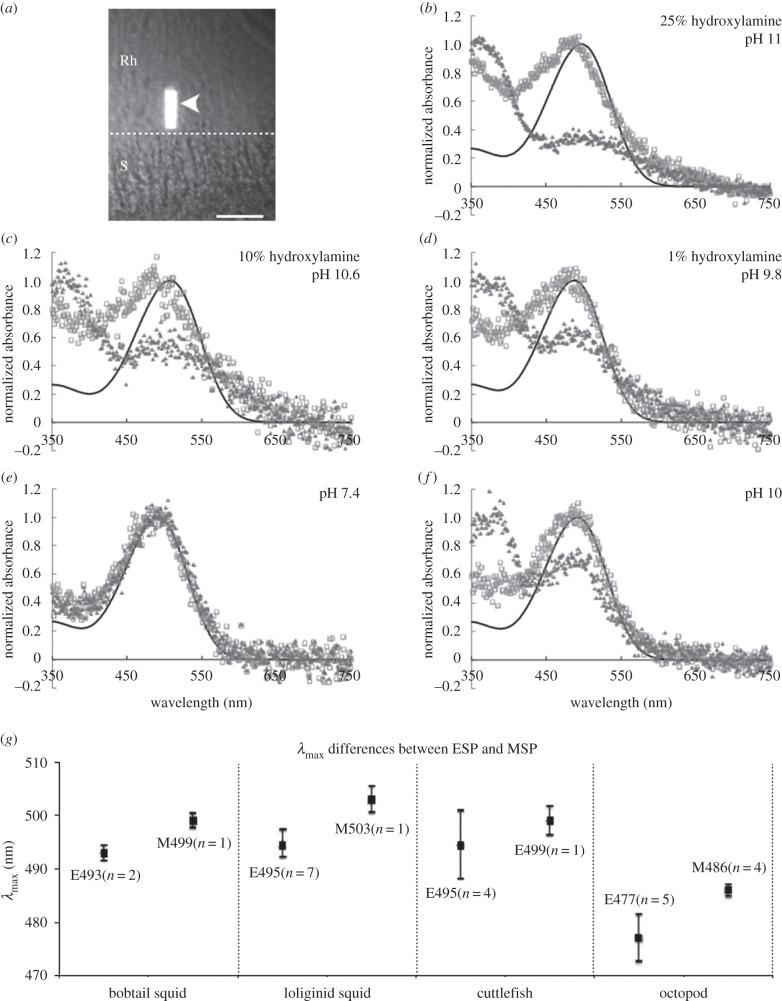
Samples of coleoid microspectrophotometric measurements using different mountants (*I. notoides*). (*a*) A sample of the MSP retinal preparation seen via an infrared image converter. Arrowhead indicates the light beam; S, screening pigment granules; Rh, rhabdominal layer. Scale bar, 20 µm. (*b–d*) Mean pre-bleached (open grey square) spectra and post-bleached spectra (dark grey triangle) in different concentrations of hydroxylamine solution. Pre-bleached spectra are overlaid with the best-fit rhodopsin template (black line). (*e*) Changes of the paired spectral curves in the standard mountant (*λ*_max_ shift from 492 to 494 nm). (*f*) Changes of the paired spectral curves in the alkaline mountant (*λ*_max_ shift from 492 to 380 nm). (*g*) *λ*_max_ differences between ESP and MSP results (mean ± 1 s.d.) in four coleoid groups. The capital E indicates *λ*_max_ measured by ESP; M indicates that using MSP; *n* indicates the number of species.

Comparisons of *λ*_max_ values obtained from initial absorbance measurements in different mountants showed no significant difference within species (e.g. *I. notoides*, less than 4 nm, *n* = 30; figure 1). However, the photo-chemical reactions of visual pigment in each of the three mountants were different, particularly the reaction speed and the associating spectral absorbance of the photo-products (figure 1).

The bleaching procedure using the diluted hydroxylamine solution (pH 9.8–11) was remarkably fast. Thirty seconds of white light irradiation sufficiently bleached visual pigments as indicated by a significant drop in the main absorbance peak at, for example, 492 nm, and a peak of photo-product appearing at short wavelengths (approx. 360 nm) (figure 1*b–d*). Irradiation using this method also caused a large area of partially bleached visual pigments near the beam, making it difficult for subsequent MSP measurements. This method was therefore rejected.

Using a standard PBS mountant, repeated exposure to bright white light for at least four 5 min periods was required for significant effect of bleaching, resulting in 2–4 nm of spectral peak shift between the paired scans (rhodopsin versus acid-metarhodopsin; figure 1*e*). After long periods of irradiation, over 90% of scans showed movement artefacts between the paired-measurements (*n* = 75), again making this method not ideal for accurate MSP.

The alkaline mountant shortened the duration of the bleaching process and revealed distinctive changes in spectra and peak positions between scan pairs. A 2 min white light irradiation was sufficient to bleach visual pigments (figure 1*f*) and the partially bleached area around the beam was reduced to approximately 50 µm diameter, allowing effective measurement of neighbouring photoreceptors. This method was therefore chosen as the best for subsequent species comparisons.

### (b) *λ*_max_ from microspectrophotometry

Using the new MSP protocol developed here (alkaline mountant), visual pigment distribution across the retina was mapped in eight coastal coleoid species (table 1; electronic supplementary material, table S1). No difference was found between visual pigment absorbance over any of the retinal areas examined within each species (electronic supplementary material, table S1). The spectral sensitivities between species can be categorized into two groups: (i) *λ*_max_ close to 485 nm in four coastal octopods (484–488 nm); and (ii) *λ*_max_ close to 500 nm in four coastal decapodiform coleoids (493–504 nm). In addition, the MSP results showed that *λ*_max_ values are consistently longer (approx. 6 nm) than ESP data where four groups of coleoids were examined with both methods (figure 1*g*). As a result, for effective comparison, previous ESP data were offset by 6 nm prior to the following analyses.

### (c) Cephalopod opsin analyses and correlation of *λ*_max_ with habitat

**Figure 2.**
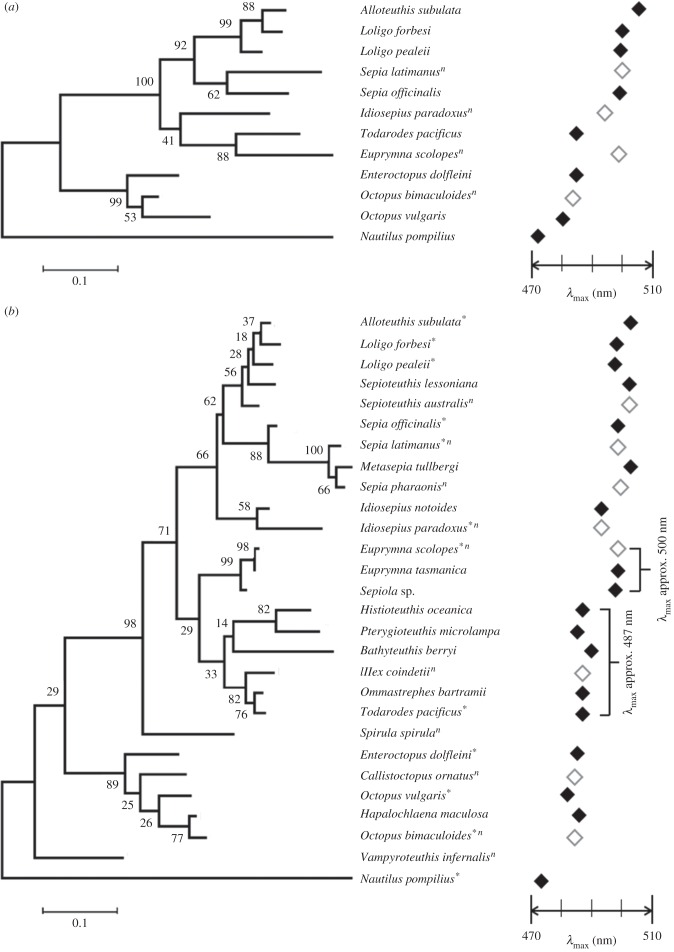
Maximum-likelihood (ML) phylogeny of cephalopod opsins. (*a*) The tree constructed using 12 species with the full-length opsin transcripts. (*b*) The tree constructed using 28 partial opsin transcripts. Both opsin trees showed that a close relationship exists between oegopsid and sepiolid opsins despite their spectral sensitivity differentiating by more than 10 nm. Asterisk next to species name indicates the full-length opsin sequence; *n* next to species name indicates no available *λ*_max_ values; closed diamonds indicate *λ*_max_ obtained from MSP or the adjusted ESP values; open diamonds indicate the predictions of *λ*_max_ values by simply averaging available *λ*_max_ data from their relatives. The bootstrap values are shown in front of the branch nodes.

Estimation of the phylogenetic signal, Pagel's *λ*, showed similar results in two opsin trees, representing a strong phylogenetic relationship where Pagel's *λ* = 0.9637 for 12 full-length opsin transcripts (test of *λ* = 1, *p* = 0.64) and 0.9247 for 28 partial transcripts (test of *λ* = 1, *p* = 0.11), respectively. In contrast with the ancestral form of visual pigment of *Nautilus* (*λ*_max_ 473 nm) [17], the spectral sensitivity of coleoids possesses some degree of bathochromatic (longer wavelength) shift (8–32 nm; figure 2). Phylogenetic linear regression showed that their *λ*_max_ changes to longer wavelengths are correlated with habitat (PGLS, *n* = 12, adjusted *R*^2^ = 0.2204, *t* = −2.17, *p* = 0.058; PGLS, *n* = 28, adjusted *R*^2^ = 0.35, *t* = −3.62, *p* < 0.002; electronic supplementary material, tables S3 and S4).

Another analysis focused on 19 decapodiform opsins which revealed a weak phylogenetic signal, Pagel's *λ* = 0.2705 (test of *λ* = 1, *p* < 0.001). However, the corresponding PGLS analysis showed again that the *λ*_max_ values are strongly correlated with habitat (PGLS, *n* = 19, adjusted *R*^2^ = 0.7176, *t* = −4.9506, *p* < 0.0002), most particularly the ambient light conditions of their habitat (table 1; electronic supplementary material, tables S3 and S4).

Both opsin trees showed that a close relationship exists between oegopsid and sepiolid opsins despite their spectral sensitivity differentiating by more than 10 nm, indicating that amino acid substitutions between these two groups could be the key sites in determining spectral tuning. A comparison of 12 full-length opsin sequence alignments revealed a high degree of conservation in both cytoplasmic and extracellular loops as well as TM1, 2, 6 and 7 (tables 2 and 3; electronic supplementary material, table S2). The numbers of amino acid sites that differed among 12 species are summed in table 2. Examining *Nautilus* first, the ratio of amino acid substitutions between the transmembrane and non-transmembrane region is close to 1 : 1 among 12 cephalopods. Comparing within the 11 coleoid species, more differences of amino acid substitutions were observed in the transmembrane regions (approx. 60%; electronic supplementary material, table S2). Along with the substitutions in the transmembrane region within the 11 coleoids, changes located in the chromophore-binding pocket versus those outside the pocket were more frequent at a ratio of 7 : 3. In addition, 28 sites in the transmembrane region were identified with more variability than the rest of the amino acid sites (table 3). Taking into account the numbers of sites where substitutions altered the amino acid polarity and the tuning model developed by Bellingham *et al.* [11], nine sites of particular importance in coleoid opsin function are suggested (tables 3 and 4).

**Table 2.** Amino acid changes among cephalopod opsins.

	among 12 cephalopods	among 11 coleoids	among 8 decapodiforms	among 3 myopsids	among 3 octopods
total numbers of AA differences	188	123	80	29	15
total numbers of AA differences in the transmembrane region	105	72	43	18	14
total numbers of AA differences in the chromophore-binding pocket	54	44	29	12	7
total numbers of AA polarity changes in the chromophore-binding pocket	26	20	12	3	3

**Table 3.** Estimates of the potential tuning sites in coleoids.

transmembrane helices	sites with high relative evolutionary rate estimated by MEGA 6	known tuning sites (Bellingham *et al.* [11])	possible tuning sites (amino acid polarity changes)
TM1	36, 50, 54		
TM2	98		
TM3	105, 113, 120, 127	127, (A127S or A127 T, 12 nm shift)	105, 127
TM4	161, 164, 165, 167	167, (A167S, −2 nm shift)	165, 167
TM5	195, 196, 202, 206, 207, 208, 210, 211, 214, 217	205 (F205 T, 0 nm shift)	195, 196, 210, 211, 214
TM6	254, 258, 268, 271, 279	270, (F270S, 5 nm shift)

**Table 4.** List of possible tuning sites in determining spectral shifts of coleoid.

group	possible tuning sites (amino acid substitutions)								
	105	127	165	167	195	196	210	211	214
Idiosepiida (*λ*_max_ 493 nm, 1 species)	M	S	M	A	P	S	M	L	I
Sepiolida (*λ*_max_ 499 nm, 1 species)	H	A	L	A	Y	A	C	F	T
Myopsida (*λ*_max_ 500 nm, 3 species)	N/Q	T	I/T	A	A/S/T	S/T	M	C/F	I/V
Sepiida (*λ*_max_ 499 nm, 2 species)	M/N	A/S	L	S	S/Y	A/V	C	F	L
Oegopsida (*λ*_max_ 487 nm, 1 species)	F	A	L	A	S	T	F	G	L
Octopoda (*λ*_max_ 485 nm, 3 species)	K	A	V	S	S/P	N/S	M	L	I/V

## 4. Discussion

The new MSP protocol established here (alkaline mountant) significantly accelerates the bleaching process and makes assessment of cephalopod spectral sensitivity more accurate. As the spectral peak of alkaline metarhodopsin appears at much shorter wavelengths separated from the main peak of the visual pigment, one major difficulty in determining an isolated spectral curve for rhodopsin is resolved. In most invertebrates, a bistable metarhodopsin is formed on exposure to light and this method may be useful in other invertebrate taxa also.

Aside from this methodological advance, the direct MSP evidence presented here indicates that the eight species of coleoid examined all possess a single visual pigment. As a result, unless the unlikely optical solution for colour vision recently suggested by Stubbs & Stubbs [35] can be proved, colour-blindness remains a common feature in all examined coastal coleoids so far.

As many coastal coleoids live in shallow waters and are under intense predatory pressure, it is perhaps surprising that they have not evolved colour vision for predatory avoidance, mating interactions or indeed their own also very aggressive predatory feeding style [1]. They are famously masters of camouflage, a strategy that presumably is used in all these necessary behavioural interactions. Aside from spending most of their time hidden, ‘dressed’ in effective camouflage, octopus and cuttlefish can easily switch coloration into high-contrast black and white patterns to emphasize their existence, startle potential threats or attract mates [1]. In addition, it has recently been suggested that coleoids have developed polarization vision and polarization signals in place of colours [36,37]. As the cephalopods were among the first animals to evolve complex visual abilities, it is fascinating to speculate that polarization vision may have evolved before colour vision and indeed in shallow water environments it has some advantages [36,37].

Bellingham *et al.* [11] developed the first spectral tuning model for coleoids. Although their model suggests that there are four main substitution sites (127, 167, 205 and 270) critical in spectral tuning (table 3), the substitution occurring at the site 205 (F205Y indicating from phenylalanine to tyrosine at site 205) was only found in one species (table 2 in [11]). Our current study also noted that F205Y was only found in one other pelagic species, *Illex coindetii*, suggesting that this site is unlikely to be critical in coleoid spectral tuning. In addition, three of the other sites proposed by Bellingham *et al.* were then further tested here to see whether the estimated *λ*_max_ values using their model are well matched with the current MSP results. Our MSP results for *S. lessoniana* are well matched with the estimated *λ*_max_ (approx. 500 nm), as are the results for the octopods (approx. 485 nm). However, a mismatch between the Bellingham *et al.* estimated *λ*_max_ and current MSP results was found in cuttlefish (Δ10 nm), bobtail squid (Δ12 nm) and pygmy squid (Δ7 nm). Along with these mismatched *λ*_max_ estimates, two other inconsistencies between our results and the Bellingham *et al.* model are identified. First, alignments of 28 opsin sequences showed that the substitution of F270S and the resulting 5 nm green-shift only occurred in *Alloteuthis subulata.* It is also not possible to explain how the other 13 coastal decapodiform coleoids studied here possess the green-shifted *λ*_max_ without the substitution F270S. Second, the substitution at site 127 and its hypothesized 12 nm shift is also problematic, particularly in decapodiforms. This substantial suggested shift underestimates the sepiolid's *λ*_max_, whereas the *λ*_max_ of pygmy squid is overestimated. Thus, it is clear that these three proposed sites of the Bellingham *et al.* model alone cannot explain the spectral changes of coleoids, indicating the existence of additional mechanisms in coleoid spectral tuning.

Both of the opsin trees generated in our analysis show a close relationship between oegopsids and sepiolids, however, their spectral sensitivities are more than 10 nm variation (figure 2). These groups contain diverse species with a broad geographical distribution. Their habitats range from coastal waters (less than 100 m) to mid-water (200–1000 m), where ambient brightness could vary over 1000 times and the spectral range or colour of the water they inhabit may vary from green to blue [6,38,39].

PGLS results also indicate that the *λ*_max_ of mid-water squid is most likely to be the result of adaptation to blue open ocean realms they inhabit and is similar to spectral tuning adaptations found in many deep-sea fishes [10,23]. This also indicates that the amino acid replacements occurring between these two coleoid groups are likely to be those responsible for spectral tuning to different habitats. To date, the full-length opsin transcripts are only available in *Todarodes pacificus* and *Euprymna scolopes*, whereas the other available opsins in these two groups contain a large portion of unsequenced opsin-coding regions. The nine amino acid substitution sites potentially critical in coleoid spectral tuning proposed in this study therefore have to be further tested when full-length opsins can be matched to corresponding MSP or ESP.

Finally, it is worth noting that Strugnell *et al.* [27] discovered a close relationship between octopod opsins, grouping coastal and deep-sea octopods in the same cluster of the opsin tree (appendix 2 in [27]). Both these results and ours suggest that the octopods respond less to changes in light environment than do squid or cuttlefish. One possible explanation for this is the generally benthic foraging behaviour of octopods that often relies on tactile and chemoreception input more than vision. In contrast to the visual predators (e.g. squid and fish) that rely on visual information [6,10], octopod vision might therefore be under reduced selection pressure, at least in terms of tuning *λ*_max_ to precisely match the dominant spectra of their realm.

In summary, all examined coastal coleoids possess a single visual pigment, indicating an inability to distinguish colours in these visual predators. Our current data also show that coleoid opsins have undergone spectral tuning in decapodiforms, whereas octopod visual pigments are not tuned to match with their dwelling light conditions. Furthermore, the low substitution rate of opsin and monochromacy of coleoids make using their opsin genes combined with spectral measurement an effective molecular marker in studying functional adaptation and evolutionary history in these remarkable creatures.

## Supplementary Material

supplementary materials

## Data accessibility

Data supporting this article are available as the electronic supplementary material.
