# Supplementary material for: Comparative visual ecology of cephalopods from different habitats
Source: Proc Biol Sci. 2016 Sep 14;283(1838):20161346. doi: 10.1098/rspb.2016.1346 (PMC5031660; doi:10.1098/rspb.2016.1346)
Supplement: supplementary materials [file rspb20161346supp1.pdf]

**Title: Comparative visual ecology of cephalopods from different habitats**

**Authors: Wen-Sung Chung & N. Justin Marshall**

**Supplementary materials**

## Microspectrophotometry (MSP) procedure

Animals were dark-adapted in aerated aquaria for at least 8 hours prior to the retinal preparation for MSP. The specimen was anesthetised in cold seawater mixed with 2% $MgCl_2$ and then decapitated. Eyes were removed under dim red illumination (690 nm LED). Retinal preparations were conducted under infrared illumination with the aid of a dissecting microscope fitted with an infrared image converter (Electrophysics, USA). The cornea and lens were removed and the eyecup was placed in 0.1 M phosphate buffer saline (PBS) (17-515DPBS, Lonza, USA) containing 6% sucrose. For large eyes (diameter 10-15 mm), each eye cup was symmetrically divided into four quadrants and three rectangular shaped retinal samples (ca. 2 x 4 mm) were selected from each quadrant. These retinal pieces were embedded in the Optimal Cutting Temperature compound (OCT) (Tissue-Tek, Sakura Finetek, USA) mixed with 10% sucrose for cryosectioning at -20$^{o}$C under dim red illumination (690 nm LED). For small eyes (diameter 2-9 mm), an entire eyeball was embedded for cryosectioning. Transverse section of the retina (12 μm thickness), were collected with a coverslip (22 x 64 mm #1, Menzel-Glaser, Germany).

Three different mounting solutions were used for MSP measurement as follows: (1) The standard mounting solution (0.1M PBS mixed with 6% sucrose, pH 7.4). (2) The alkaline mounting solution is 0.1M PBS mixed with 6% sucrose and its pH value is adjusted to 10

using 0.1 M NaOH solution. pH was measured with a FiveEasy pH meter (Mettler Toledo, United Kingdom). (3) Hydroxylamine solution (50% w/v solution, Merck, Germany) was diluted into three concentrations, 25% (pH 11), 10% (pH 10.6), and 1% (pH 9.8) respectively.

A drop of the mounting solution was placed on a freshly cut retinal slice. The preparation was covered with a circular No. 0 glass coverslip (10 mm diameter #0, Chance Propper, United Kingdom). The edges of the top coverslip were then sealed with silicone vacuum grease to prevent dehydration. With the aid of dim red illumination, the preparation was then positioned on the stage of the microscope for MSP measurement. Other than brief exposure to dim red light, all procedures were performed using infrared illumination and an image converter in a constant temperature (22$^{o}$C) dark room.

**MSP operation and data analysis**

Operation of MSP followed a standard protocol developed for vertebrate or invertebrate photoreceptors [1-3]. The measuring light beam was set to a size of 2 x 15 μm and placed parallel to the long axis of the rhabdome (main text Figure 1). The relatively large size of rhabdome (ca. 4x120 - 3x270 μm) made this easy. Baseline and sample scans were made from tissue-free and cellular regions of the preparation respectively. The scanning spectral range was between 300 and 800 nm with a measurement taken every 1 nm throughout 2 passes, a downward long-to-short wavelength scan and an upward short-to-long wavelength scan. If both scans revealed no obvious distortions and the absorbance spectra were flat for 100 nm beyond the wavelength at which the long wavelength limb first dropped to zero absorption, two curves were then averaged and saved. Subsequently, the visual pigment was bleached using a white light beam (ca. 2 x 15 μm) and the absorption spectrum of photo-

product was measured using the same MSP protocol. The bleaching process was repeated in some cases until effective visual pigment bleaching occurred.

Baseline and sample data were converted to absorbance values at each wavelength. Best fit visual pigment nomograms were used to determine the λmax of each sample following the methods developed by MacNichol [4], Govardovskii et al [5] and Hart et al [3]. Absorbance spectra from each measurement that satisfied the selection criteria established by Hart, Partridge [3] were accepted and these data in the sampling retinal region were averaged.

**Spectral sensitivity and photochemistry reactions under different mountants**

Comparisons of λmax values obtained from different mountants showed no significant difference within species, representing a slight variation of the averaged spectral sensitivity (< 4 nm). However, addition of irradiation made photo-chemical reactions of visual pigment behave differently in three mountants, particularly the reaction speed and changes of waveform due to different types of photo-product (main text Figure 1).

**Hydroxylamine solution**

The bleaching procedure using hydoroxylamine solution was remarkably fast and sensitive. The rate of reaction depended on concentration of hydroxylamine. Given a short period of 30-second irradiation of a white light beam, high concentration hydroxylamine solutions were capable of turning an extended range of retinal slice insensitive to light, forming a patch of irreversible photo-product over 4 mm diameter adjust to the light beam in 25% solution and around 2 mm diameter in 10% one. By contrast, with further diluted hydroxylamine solution (1%), 30-second white light irradiation sufficiently bleached visual pigments, resulting that the spectral peak of visual pigment dropped and consequently another peak of

the photo-product raised at short wavelengths (approx. 360 nm) (main text Figure 1b-d). Also, irradiation caused amounts of partially-bleached visual pigments near the light beam. The partially-bleached MSP curve can be visually identified by the two-peak broad spectrum, resulting disadvantages which the subsequent MSP measurements were therefore frequently rejected due to the selection criteria and exclusive in further analysis [3]. In most of measurements, repeated bleaching procedures caused the retinal slice completely lost photosensitivity within a sum of 5-minute white light irradiation.

Addition of hydroxylamine instantaneously reacts with the free-chromophore and destroys acid metarhodopsin with the result that the released chromophores from both rhodopsin and metarhodopsin are converted to retinene-oxime (photo-product λmax approx. 380 nm) [6]. This could explain that a short period of light exposure caused rapid loss of photosensitivity of an entire retinal slice. Also, the production of partially-bleached visual pigments after a short period of illumination (mainly due to the light exposure during MSP measurement) often resulted the distorted spectral curve where the long wave limb raised up in the following MSP measurement. This feature, the tilted long-wave limb of the curve and the widen half bandwidth, caused the best fit curve mismatched with the template of visual pigment suggested by Govardovskii, Fyhrquist [5], rendering suspected artefacts for further analyses.

**Standard mountant (pH7.4)**

In standard mountant, repeated exposure to bright white light for at least four five-minute periods was required for significant effect of bleaching. The spectrum of the mixture of photo-product (acid-metarhodopsin) and rhodopsin became gradually flattened while its maximal sensitive peak remained nearly at the same spectral peak at the λmax of the

rhodopsin (i.e. pre-bleaching λmax 492 nm versus post-bleaching λmax 494 nm in *I. notoides*; 502 versus 505 nm in *S. lessoniana*) (main text Figure 1e). The whole bleaching process usually took 15-25 minutes. In 92% of scans (n = 75), the post-bleaching spectrum did not lie near the pre-bleaching spectrum at long wavelengths, particularly beyond 600 nm. With this measuring artefact, these mismatch-paired measurements were exclusive in further analysis.

**Alkaline mountant (pH10)**

The alkaline mountant shortened the duration of the bleaching process and revealed distinctive changes in waveform between scan pairs (pre- and post-bleached scans) (main text Figure 1f). In 87% of scans, a 2-minute white light beam exposure was sufficient to generate a distinctive separation of spectral peaks, resulting a second peak of the photo-product (alkaline-metarhodopsin) appeared at short wavelengths (approx. 380 nm) (main text Figure 1f). Another advantage is to restrict the partially-bleached area within a small range (approx. 50 μm), allowing to measure neighbouring photoreceptors. Along with these advantages in measuring cephalopod visual pigment, investigating the selected cephalopods in this study was adopted this new protocol therefore.

**Phylogenetic data:**

TreeBASE access number: 19730 (http://purl.org/phylo/treebase/phylows/study/TB2:S19730)

| Group | Species | Retinal Quadrant | λmax (nm) |
|---|---|---|---|
| Cuttlefish | *Sepia plangon* (n=3, ML: 10-24 mm) | AD | 498 ± 1.8 (N=29) |
| | | AV | 498 ± 2.2 (N=29) |
| | | PD | 498 ± 2.7 (N=25) |
| | | PV | 500 ± 3.2 (N=29) |
| Octopus | *Callistoctopus dierythraeus* (n=3, ML: 65-80 mm) | AD | 484 ± 1.9 (N=23) |
| | | AV | 487 ± 2.5 (N=21) |
| | | PD | 488 ± 1.8 (N=21) |
| | | PV | 487 ± 1.8 (N=18) |
| | *Hapalochlaena maculosa* (n=4, ML: 8-13 mm) | AD | 486 ± 3.1 (N=18) |
| | | AV | 485 ± 2.4 (N=18) |
| | | PD | 485 ± 2.2 (N=22) |
| | | PV | 485 ± 1.7 (N=21) |
| | *Octopus australis* (n=6, ML: 10-15 mm) | AD | 485 ± 1.3 (N=15) |
| | | AV | 484 ± 1.2 (N=15) |
| | | PD | 485 ± 1.1 (N=15) |
| | | PV | 485 ± 1.3 (N=15) |
| | *Octopus tetricus* (n=3, ML: 55-65 mm) | AD | 487 ± 2.7 (N=20) |
| | | AV | 488 ± 2.3 (N=20) |
| | | PD | 484 ± 4.6 (N=22) |
| | | PV | 487 ± 2.3 (N=20) |
| Squid | *Euprymna tasmanica* (n=3, ML: 6-10 mm) | AD | 498 ± 1.4 (N=25) |
| | | AV | 498 ± 1.3 (N=25) |
| | | PD | 498 ± 1.1 (N=25) |
| | | PV | 498 ± 1.4 (N=25) |
| | *Idiosepius notoides* (n=12, ML: 6-10 mm) | AD | 493 ± 2.5 (N=24) |
| | | AV | 492 ± 2.1 (N=19) |
| | | PD | 493 ± 1.4 (N=27) |
| | | PV | 492 ± 1.4 (N=30) |
| | *Sepioteuthis lessoniana* (n=5, ML: 22-52 mm) | AD | 505 ± 2.5 (N=21) |
| | | AV | 504 ± 1.5 (N=21) |
| | | PD | 502 ± 1.6 (N=35) |
| | | PV | 503 ± 2.0 (N=21) |

**table S1** Microspectrophotometrical data of four retinal quadrants in eight cephalopods. Mantle length (ML) (A: anterior; P: posterior; D: dorsal; V: ventral). Values are mean ± 1S.D.

TM1

| amino acid site | 10 | 20 | 30 | 40 | 50 |
|---|---|---|---|---|---|
| *Todarodes pacificus* | **MGRDLRDNET** | **WWYNPSIVVH** | **PHWREFDQVP** | **DAVYYSLGIF** | **IGICGIIGCG** |
| *Idiosepius paradoxus* | .A.GTP.... | .....YME.. | S..KQ..... | .....C.... | .......... |
| *Euprymna scolopes* | ....IP.... | .......... | .......... | .....G.... | ......?... |
| *Alloteuthis subulata* | -------... | .....YMDI. | S..KQ..... | A......... | .A.......A |
| *Doryteuthis pealeii* | ....IP.... | .....YMEIN | S..KQ..... | A......... | .........V |
| *Loligo forbesii* | ....IP.... | .....YMDI. | ...KQ..... | A......... | .A.......V |
| *Sepia latimanus* | ....IP.... | .....TME.. | ...IQ..... | .......... | ......V... |
| *Sepia officinalis* | ....IP.... | .....TME.. | ...KQ.N... | .......... | .........T |
| *Entroctopus dofleini* | VESTTLV.Q. | .....TVDI. | ...AK..PI. | ......V... | ..VV....IL |
| *Octopus bimaculoides* | VESSTLV.Q. | .....TVDI. | ...AK..PI. | ......V... | ..VV....IF |
| *Octopus vulgaris* | VESTTLV.Q. | .....TVDI. | ...AK..PI. | ..I...V... | ..VV....IF |
| *Nautilus pompilius* | TTI.PENITS | V.D.EDVFI. | ...KH.PG.. | ..AH.IV... | .TVV..C.VM |

TM1 TM2

| amino acid site | 60 | 70 | 80 | 90 | 100 |
|---|---|---|---|---|---|
| *Todarodes pacificus* | **GNGIVIYLFT** | **KTKSLQTPAN** | **MFIINLAFSD** | **FTFSLVNGFP** | **LMTISCFLKK** |
| *Idiosepius paradoxus* | .......... | .......... | .......... | .......... | .......... |
| *Euprymna scolopes* | .......... | .......... | .......... | .......... | .......... |
| *Alloteuthis subulata* | .......... | .......... | .......... | .......... | M........H |
| *Doryteuthis pealeii* | .......... | .......... | .......... | .......... | .........Y |
| *Loligo forbesii* | ...V...... | .......... | .......... | .......... | .......M.Y |
| *Sepia latimanus* | .......... | .......... | .......... | .......... | .......I.. |
| *Sepia officinalis* | .......... | .......... | .......... | .......... | .......I.. |
| *Entroctopus dofleini* | ...V.....S | .......... | .......M.. | LS..AI.... | .K...A.M.. |
| *Octopus bimaculoides* | ...V.....S | .......... | .......M.. | LS..AI.... | .K...A.M.. |
| *Octopus vulgaris* | ...V.....S | .......... | .......M.. | LS..AI.... | .K...A.M.. |
| *Nautilus pompilius* | ...V...I.S | R....R.... | .......L.. | L...A..... | .LS..S.Q.. |

TM3 TM4

| amino acid site | 110 | 120 | 130 | 140 | 150 |
|---|---|---|---|---|---|
| *Todarodes pacificus* | **WIFGFAACKV** | **YGFIGGIFGF** | **MSIMTMAMIS** | **IDRYNVIGRP** | **MAASKKMSHR** |
| *Idiosepius paradoxus* | ....M..... | .......... | ......S... | .......... | .......N.. |
| *Euprymna scolopes* | .V..H..... | .........L | .......... | .......... | .....H.... |
| *Alloteuthis subulata* | .V..Q..... | ..L......L | T.....T... | ........R. | .S........ |
| *Doryteuthis pealeii* | .V..N..... | ..L......L | ......T... | .......... | .S........ |
| *Loligo forbesii* | .V..N..... | ..L......L | ......T... | .......... | .S........ |
| *Sepia latimanus* | .V..N..... | .........L | .......... | .......... | .......... |
| *Sepia officinalis* | .V..M..... | .........L | ......S... | .......... | .......... |
| *Entroctopus dofleini* | ....KV..QL | ..LL...... | ...N...... | .......... | .......... |
| *Octopus bimaculoides* | ....KV..QL | ..LL...... | ...N...... | .......... | .......... |
| *Octopus vulgaris* | ....KV..QL | ..LL...... | ...N...... | .......... | .......... |
| *Nautilus pompilius* | ....QT..EL | ..LA.....L | ...N...... | ........A. | ..V..R...K |

TM4 | TM5

| amino acid site | | 160 | | 170 | | 180 | | 190 | | 200 |
|---|---|---|---|---|---|---|---|---|---|---|
| *Todarodes pacificus* | **R A F I M I I F V** | **W** | **L W S V L W A I G** | **P** | **I F G W G A Y T L** | **E** | **G V L C N C S F D** | **Y** | **I S R D S T T R S** | **N** |
| *Idiosepius paradoxus* | . . . L . . . . . | . | . . . T M . . . . | . | . . . . . . . A . | . | . . . . . . . . . | . | . . . . P S . . . | . |
| *Euprymna scolopes* | . . . . . . . . . | . | . . . . . . . . . | . | . . . . . . . . . | . | . . . . . . . . . | . | . T . . Y A . . . | . |
| *Alloteuthis subulata* | K . . . . . V . . | . | I . . T I . . . . | . | . . . . . . . Q . | . | . . . . . . . . . | . | . T . . A S . . . | . |
| *Doryteuthis pealeii* | K . . . . . . . . | . | I . . T T . . . . | . | . . . . . . . S . | . | . . . . . . . . . | . | . . . . . S . . . | . |
| *Loligo forbesii* | K . . . . . . . . | . | I . . T I . . . . | . | . . . . . . . . . | . | . . . . . . . . . | . | . T . . T . . . . | . |
| *Sepia latimanus* | . . S L . . . . . | . | M . . T . . S . . | . | . . . . . . . V . | . | . . . . . . . . . | . | . T . . Y V . . . | . |
| *Sepia officinalis* | . . . L . . . . . | . | M . . T . . S . . | . | . . . . . . . V . | . | . . . . . . . . . | . | . T . . . A . . . | . |
| *Entroctopus dofleini* | . . . L . . . . . | . | M . . I V . S V . | . | V . N . . . . V P | . | . I . T S . . . . | . | L . T . P S . . . | F |
| *Octopus bimaculoides* | . . . L . . . . . | . | I . . I V . S V . | . | V . N . . . . V P | . | . I . T S . . . . | . | L . T . . S . . . | F |
| *Octopus vulgaris* | . . . L . . . . . | . | I . . I V . . V . | . | V . N . . . . V P | . | . I . T S . . . . | . | L . T . . N . . . | F |
| *Nautilus pompilius* | K . . . . . . S . | . | I . A A V . T L P | . | L . . . . . . I P | . | . F Q T S . T . . | . | L T . N N Y F . . | Y |

TM5 | TM6

| amino acid site | | 210 | | 220 | | 230 | | 240 | | 250 |
|---|---|---|---|---|---|---|---|---|---|---|
| *Todarodes pacificus* | **I L C M F I L G F** | **F** | **G P I L I I F F C** | **Y** | **F N I V M S V S N** | **H** | **E K E M A A M A K** | **R** | **L N A K E L R K A** | **Q** |
| *Idiosepius paradoxus* | . I . . Y . . . . | M | L . . I . . . . . | . | . . . . . . . . . | . | . . . . . . . . . | . | . . . . . . . . . | K |
| *Euprymna scolopes* | . I . . Y V F A . | C | F . . T . . L . . | . | . . . . . . . . . | . | . . . . . . . . . | . | . . . . . . . . . | . |
| *Alloteuthis subulata* | . V . . Y . F A . | M | F . . V V . . . . | . | . . . . . . . . . | . | . . . . . . . . . | . | . . . . . . . . . | . |
| *Doryteuthis pealeii* | . V . . Y L F A . | M | C . . I V . . . . | . | . . . . . . . . . | . | . . . . . . . . . | . | . . . . . . . . . | . |
| *Loligo forbesii* | . . . . Y . F A . | M | C . . V V . . . . | . | . . . . . . . . . | . | . . . . . . . . . | . | . . . . . . . . . | . |
| *Sepia latimanus* | . V . . Y . F A . | C | F . . . . . . . . | . | . . . . . . . . . | . | . . . . . . . . . | . | . . . . . . . . . | . |
| *Sepia officinalis* | . V . . Y . F A . | C | F . . . . . . . . | . | . . . . . A . . . | . | . . . . . . . . . | . | . . . . . . . . . | . |
| *Entroctopus dofleini* | . . . . Y F C . . | M | L . . I . . A . . | . | . . . . . . . . . | . | . . . . . . . . . | . | . . . . . . . . . | . |
| *Octopus bimaculoides* | . . . . Y F C . . | M | L . . I . . A . . | . | . . . . . . . . . | . | . . . . . . . . . | . | . . . . . . . . . | . |
| *Octopus vulgaris* | . . . . Y F M . . | M | L . V V . . A . . | . | . . . . . . . . . | . | . . . . . . . . . | . | . . . . . . . . . | . |
| *Nautilus pompilius* | V . . L Y L F . . | I | T . V T . . A I . | . | . F . F K A . A D | . | . . . . . K . . . | K | M . . . . I . A G | A |

TM6 | TM7

| amino acid site | | 260 | | 270 | | 280 | | 290 | | 300 |
|---|---|---|---|---|---|---|---|---|---|---|
| *Todarodes pacificus* | **A G A N A E M R L** | **A** | **K I S I V I V S Q** | **F** | **L L S W S P Y A V** | **V** | **A L L A Q F G P L** | **E** | **W V T P Y A A Q L** | **P** |
| *Idiosepius paradoxus* | . . . . . . . K . | . | . . . . . . . T . | . | M . . . . . . . . | . | . . . . . . . . P | . | . . . . . . . . . | . |
| *Euprymna scolopes* | . . . S . . . K . | . | . . . . . . . T . | . | . . . . . . . . I | . | . . . . . . . . . | . | . . . . . . . . . | . |
| *Alloteuthis subulata* | . . . S . . . K . | . | . . . . . . . T . | S | . . . . . . . . I | . | . . . . . . . . I | . | . . . . . . . . . | . |
| *Doryteuthis pealeii* | . . . S . . . K . | . | . . . . . . . T . | . | . . . . . . . . I | . | . . . . . . . . . | . | . . . . . . . . . | . |
| *Loligo forbesii* | . . . . . . . K . | . | . . . . . . . T . | . | . . . . . . . . . | . | . . . . . . . . I | . | . . . . . . . . . | . |
| *Sepia latimanus* | . . . . . . . . . | P | . . . . . . . . . | . | . . . . . . . . . | . | . . . . . . . . I | . | . . . . . . . . . | . |
| *Sepia officinalis* | . . . S . . . K . | . | . . . . . . . T . | . | . . . . . . . . . | . | . . . . . . . . I | . | . . . . . . . . . | . |
| *Entroctopus dofleini* | . . . S . . . K . | . | . . . M . . I T . | . | M . . . . . . . I | I | . . . . . . . . A | . | . . . . . . . E . | . |
| *Octopus bimaculoides* | . . Q S . . . K . | . | . . . M . . I T . | . | M . . . . . . . I | I | . . . . . . . . A | . | . . . . . . . E . | . |
| *Octopus vulgaris* | . . Q S . . . K . | . | . . . M . . I T . | . | M . . . . . . . . | I | . . . . . . . . A | . | . . . . . . . E . | . |
| *Nautilus pompilius* | S E Q R . . . K I | . | . . . M I . I T . | . | . . . . T . . . . | . | . M . G . . . . P | . | . . . . . . S E V | . |

TM7

| amino acid site | 301–309 | 310 | 311–319 | 320 | 321–329 | 330 | 331–339 | 340 | 341–349 | 350 |
|---|---|---|---|---|---|---|---|---|---|---|
| *Todarodes pacificus* | **VMFAKASAI** | **H** | **NPMIYSVSH** | **P** | **KFREAISQT** | **F** | **PWVLTCCQF** | **D** | **DKETEDDKD** | **A** |
| *Idiosepius paradoxus* | ......... | . | ......... | . | ......AAQ | . | ..I.....Y | . | E..I...R. | . |
| *Euprymna scolopes* | ......... | . | ......... | . | .......N. | . | ..I....T. | . | E......R. | . |
| *Alloteuthis subulata* | ......... | . | ......... | . | ......ASN | . | ..I.....Y | . | E..I..... | . |
| *Doryteuthis pealeii* | ......... | . | ......... | . | ......ASN | . | ..I...... | . | E..I..E.. | . |
| *Loligo forbesii* | ......... | . | ......... | . | ....R.ASN | . | ..I.....Y | . | E..I..... | . |
| *Sepia latimanus* | ......... | . | ..L...... | . | ...G..ADN | . | ..IIA..R. | . | E..V..E.. | . |
| *Sepia officinalis* | ......... | . | ..L...... | . | ......AEN | . | ..IIS.... | . | E..V..... | . |
| *Entroctopus dofleini* | .L....... | . | ..IV..... | . | ......QT. | . | ..L...... | . | E..C..AN. | . |
| *Octopus bimaculoides* | .L....... | . | ..IV..... | . | ......QN. | . | ..L...... | N | E..C..ANE | . |
| *Octopus vulgaris* | .L....... | . | ..IV..... | . | ......QN. | . | ..L...... | N | E..C..AN. | . |
| *Nautilus pompilius* | ........M | . | ..IV.AL.. | . | ......NER | . | .FLIS..A. | . | E...AEPEE | S |

| amino acid site | 351–359 | 360 | 361–369 | 370 | 371–379 | 380 | 381–389 | 390 | 391–399 | 400 |
|---|---|---|---|---|---|---|---|---|---|---|
| *Todarodes pacificus* | **ETEIPAGES** | **S** | **DAAPSADAA** | **Q** | **MKEMMAMMQ** | **K** | **MQQQQAAYP** | **P** | **QGYAPPPQG** | **Y** |
| *Idiosepius paradoxus* | .A...DS.Q | T | GGGGESVD. | A | QMKE.MA.M | Q | KM.A.Q.AY | . | PQG.Y.... | . |
| *Euprymna scolopes* | .A....S.Q | T | QETSPT... | . | V........ | . | ..A...QQQ | A | AYPPQGYPP | Q |
| *Alloteuthis subulata* | .A....A.Q | . | GGESVDA.Q | M | KEM.AM.QK | M | QA...QQPA | Y | PPQGY.... | . |
| *Doryteuthis pealeii* | .A....S.Q | . | GGESADA.Q | M | KEM.AM.QK | M | QA..A.QPA | Y | PPQGY.... | . |
| *Loligo forbesii* | .A......Q | . | GGETADA.Q | M | KEM.AM.QK | M | QA...QQPA | Y | PPQGY.... | . |
| *Sepia latimanus* | ......A.Q | . | GGGGGES.D | A | AQMKEMVAM | M | QKM..QQAA | Y | PPQGGY.PQ | G |
| *Sepia officinalis* | ......A.Q | . | GGETADA.Q | M | KEM.AM.QK | M | Q...A.YP. | Q | GA.P.QGGY | P |
| *Entroctopus dofleini* | .E.VV.S.R | G | GESRD.AQM | K | EMMA.MQKM | Q | A..AAYQP. | . | PPQGY.... | . |
| *Octopus bimaculoides* | .Q.VAPS.G | G | GGESADA.Q | M | KEM.AM.QK | M | QA..A.YQQ | . | PPQGY.... | . |
| *Octopus vulgaris* | .A.VAPS.G | G | GGGE.V... | . | ......... | . | ..A...... | Q | PPPQGY.PQ | G |
| *Nautilus pompilius* | K.DDMRD.. | . | TMSNIS.GG | . | VEMSTRGRR | G | GADTRYNDR | G | DMGVSNGEI | I |

| amino acid site | 401–409 | 410 | 411–419 | 420 | 421–429 | 430 | 431–439 | 440 | 441–449 | 450 |
|---|---|---|---|---|---|---|---|---|---|---|
| *Todarodes pacificus* | **PPQGYPPQG** | **Y** | **PPQGYPPQG** | **Y** | **PPPPQGAPP** | **Q** | **GAPPAAPPQ** | **G** | **VDNQAYQA-** | **-** |
| *Idiosepius paradoxus* | .......PP | A | GYPPP.AGY | P | ...AGYP.. | P | A-------- | - | --------- | - |
| *Euprymna scolopes* | GYPPP.QGY | P | .QGYP.QGA | . | ..QGYPP.Q | G | AP.Q....E | . | ........- | - |
| *Alloteuthis subulata* | ..PPPQGYP | P | QGYPPQGYP | P | QGY.PPPQG | P | PPQGPP.QA | A | PPQGVD--- | - |
| *Doryteuthis pealeii* | ..PPPQGYP | P | QGYPP.QGY | P | .QGYPPPQG | P | PPQGPP.QA | A | PPQGVDNQA | - |
| *Loligo forbesii* | ..PPPQGYP | P | QGYPPQGYP | P | QGY.PPPQG | P | PPQGPP.QA | A | PPQGVDNQA | Y |
| *Sepia latimanus* | GYPPQGGYP | P | QGYPP.A.. | . | ..AQGYP.A | . | .Y..QGY.P | P | QGAPPQG.P | P |
| *Sepia officinalis* | .QGYP..PA | Q | GGYPPQGYP | P | ..QGYPPAQ | G | YP.QGY..P | Q | GAPPQGAPP | Q |
| *Entroctopus dofleini* | ....AY.PP | Q | GYPPQGYPP | Q | GY....Y.. | . | ....QVEAP | Q | GAPPQGVDN | Q |
| *Octopus bimaculoides* | ....AY.PQ | G | Y.PQGAYPP | Q | GY....Y.. | . | ....QGDAT | Q | GAPPQGVDN | Q |
| *Octopus vulgaris* | Y.PQGY.PQ | G | Y.PQGAYPP | Q | GY....Y.. | . | ....QGDAP | Q | GAPPQGVDN | Q |
| *Nautilus pompilius* | RDLLNAFVN | V | VGAQK.Q.P | S | TVSVAMPTI | P | TYL.PMY.S | H | GYYPPPP.H | Y |

**table S2** Alignment of 12 full-length opsin-coding amino acid sequences using the multiple sequence alignment (MUSCLE) method. Amino acid identity across species is indicated by a dot (.); (-) indicates unavailable amino acids.

| Species | λmax (nm) | Habitat | Dominant light sources during forage | Max living depth (m) | Averaged distribution depth (m) |
|---|---|---|---|---|---|
| *Idiosepius notoides* | 493 | 1 | 1 | 20 | 10 |
| *Idiosepius paradoxus* | 493* | 1 | 1 | 20 | 10 |
| *Euprymna tasmanica* | 499 | 1 | 2 | 80 | 40 |
| *Euprymna scolopes* | 500* | 1 | 2 | 200 | 100 |
| *Sepiola sp.* | 498 | 1 | 2 | 150 | 76 |
| *Alloteuthis subulata* | 505 | 1 | 2 | 500 | 275 |
| *Loligo forbesii* | 500 | 1 | 2 | 700 | 375 |
| *Doryteuthis pealeii* | 499 | 1 | 2 | 390 | 195 |
| *Sepioteuthis australis* | 503* | 1 | 2 | 70 | 35 |
| *Sepioteuthis lessoniana* | 503 | 1 | 2 | 100 | 50 |
| *Sepia pharaonis* | 500* | 1 | 2 | 130 | 70 |
| *Metasepia tullbergi* | 506 | 1 | 2 | 86 | 45 |
| *Sepia officinalis* | 499 | 1 | 2 | 200 | 125 |
| *Sepia latimanus* | 499* | 1 | 2 | 30 | 15 |
| *Spirula spirula* | NA | 2 | 3 | 1750 | 1000 |
| *Bathyteuthis berryi* | 490 | 2 | 3 | 1200 | 1000 |
| *Pterygioteuthis microlampas* | 486 | 2 | 3 | 800 | 425 |
| *Histioteuthis oceanica* | 486 | 2 | 3 | 1000 | 750 |
| *Ommastrephes bartramii* | 488* | 2 | 2 | 500 | 250 |
| *Todarodes pacificus* | 488 | 2 | 2 | 500 | 250 |
| *Illex coindetii* | 488* | 2 | 2 | 600 | 300 |
| *Vampyroteuthis infernalis* | NA | 2 | 3 | 1200 | 900 |
| *Callistoctopus ornatus* | 487 | 1 | 1 | 80 | 40 |
| *Entroctopus dofleini* | 486 | 1 | 1 | 1500 | 750 |
| *Octopus vulgaris* | 481 | 1 | 1 | 100 | 50 |
| *Hapalochlaena maculosa* | 485 | 1 | 1 | 50 | 25 |
| *Octopus bimaculoides* | 487 | 1 | 1 | 50 | 25 |
| *Nautilus pompilius* | 473 | 2 | 3 | 750 | 400 |

**table S3** Dataset used in phylogenetic linear regression (PGLS) analyses. * indicates that the predictions of λmax values are inferred using the averaged λmax of their relatives obtained by the MSP and the adjusted ESP results. For the habitat, 1 = coastal waters, 2 = mid water.

For dominant light sources during forage, 1 = sunlight, 2 = partial sunlight and bioluminescence, 3 = dominant bioluminescence.

| Species numbers | Predictor valuables | *t-value* | *p* |
|---|---|---|---|
| 28 cephalopods | Habitat | -3.6151 | **0.0016** |
| | Dominant light source during forage | 1.8685 | **0.0757** |
| | Maximum living depth | 0.14 | 0.8899 |
| | Averaged distribution depth | -0.2857 | 0.7779 |
| 19 decapodiform coleoids | Habitat | -4.9506 | **0.0002** |
| | Dominant light source during forage | 2.2561 | **0.0406** |
| | Maximum living depth | 0.0629 | 0.9508 |
| | Averaged distribution depth | -0.3003 | 0.7684 |

**table S4** The results of PGLS tests for the two different opsin phylogenies. The significance levels are shown in bold.

References

[1] Hart, N.S. 2004 Microspectrophotometry of visual pigments and oil droplets in a marine bird, the wedge-tailed shearwater *Puffinus pacificus*: topographic variations in photoreceptor spectral characteristics. *J Exp Biol* **207**, 1229-1240. (doi:Doi 10.1242/Jeb.00857).
[2] Cronin, T.W. & Marshall, N.J. 1989 Multiple spectral classes of photoreceptors in the retinas of gonodactyloid stomatopod crustaceans. *Journal of comparative physiology. A, Sensory, neural, and behavioral physiology* **166**, 261-275. (doi:10.1007/bf00193471).
[3] Hart, N.S., Partridge, J.C. & Cuthill, I.C. 1998 Visual pigments, oil droplets and cone photoreceptor distribution in the European starling (*Sturnus vulgaris*). *J Exp Biol* **201**, 1433-1446.
[4] MacNichol, E.F., Jr. 1986 A unifying presentation of photopigment spectra. *Vision Res* **26**, 1543-1556. (doi:10.1016/0042-6989(86)90174-4).
[5] Govardovskii, V.I., Fyhrquist, N., Reuter, T., Kuzmin, D.G. & Donner, K. 2000 In search of the visual pigment template. *Vis Neurosci* **17**, 509-528. (doi:10.1017/S0952523800174036).

[6] Hubbard, R. & St George, R.C.C. 1958 The rhodopsin system of the squid. *J Gen Physiol* **41**, 501-528. (doi:10.1085/Jgp.41.3.501).
